# Hypothalamic hypopituitarism secondary to suprasellar metastases from small cell lung cancer: a case report and review of the literature

**DOI:** 10.1186/s13256-018-1871-0

**Published:** 2018-11-18

**Authors:** Ryohei Ono, Ryoji Ito, Keiko Nakagawa, Shinichi Teshima, Izumi Kitagawa, Hideyasu Sugimoto

**Affiliations:** 1 Department of General Internal Medicine, Shonan Kamakura General Hospital, 1370-1 Okamoto, Kamakura city, Kanagawa 247-8533 Japan; 2 Department of Diagnostic Pathology, Shonan Kamakura General Hospital, 1370-1 Okamoto, Kamakura city, Kanagawa 247-8533 Japan; 3 Department of Respiratory Medicine, Shonan Kamakura General Hospital, 1370-1 Okamoto, Kamakura city, Kanagawa 247-8533 Japan

**Keywords:** Hypothalamic hypopituitarism, Small cell lung cancer, Hormone replacement therapy, Pan-hypopituitarism, Suprasellar, Recessus infundibuli, Metastasis

## Abstract

**Background:**

Metastasis to the pituitary gland is an infrequent clinical problem, and the symptoms caused by metastases have been reported in only 2.5–18.2% of the cases. However, metastasis to the suprasellar lesion has rarely been reported in the literature. To the best of our knowledge, only nine cases of hypothalamic hypopituitarism due to metastases of solid tumors have been reported in English-language journals.

**Case presentation:**

A 67-year-old Japanese man presented to our hospital with generalized weakness, lethargy, and weight loss. Laboratory data showed hypoglycemia together with low thyroid-stimulating hormone and free thyroxine. We suspected hypopituitarism and performed imaging of the head, which revealed multiple tumors, one of which was in the suprasellar region. Computed tomography of the chest showed a tumor shadow, and a bronchoscopic biopsy pathologically showed small cell lung cancer. Hormone profiling demonstrated hypothalamic pan-hypopituitarism. We diagnosed hypothalamic hypopituitarism secondary to metastases from the primary lung cancer and initiated radiation, chemotherapy, and hormone replacement, but the patient died 10 months later.

**Conclusions:**

We report a case of a 67-year-old man with hypothalamic hypopituitarism secondary to a suprasellar metastasis from a primary small cell lung cancer, and we review ten cases of hypothalamic hypopituitarism due to metastases, including our patient. Recognizing hypopituitarism can be challenging, especially in the elderly, whose symptoms such as lethargy and visual decline may be mistaken for the natural aging process. In patients with established metastatic conditions, the symptoms may be wrongly attributed to malignancy or to the side effects of therapy. When a patient is suspected of having hypopituitarism, a hormone load test can help to diagnose the type of hypopituitarism. It is important to evaluate the brain and the whole body to confirm whether metastasis and primary cancer exist. Because the mortality rate is very high, aggressive intervention for both diagnosis and therapy is required in cases of hypothalamic hypopituitarism secondary to tumor metastasis.

## Background

Metastasis to the pituitary gland is an infrequent clinical problem, and the symptoms caused by the metastases are reported in only 2.5–18.2% of the cases [1]. The clinical presentation of hypopituitarism is often insidious, being characterized by nonspecific manifestations, such as weight gain, fatigue, low muscle strength, bradypsychism, hypotension, or intolerance to cold. Hypopituitarism is a clinical condition characterized by decreased secretion of one or more hormones. This dysfunction can be caused by primary pituitary disease, hypothalamic disease causing secretory deficiency of hypothalamic releasing factors (releasing hormones), interruption of pituitary stalk, and extrasellar disorders [2].

However, in the literature, metastasis to the suprasellar region has rarely been reported. To the best of our knowledge, only nine cases of hypothalamic hypopituitarism due to metastases of solid tumors have been reported in English-language journals to date. We report a case of a 67-year-old man with hypothalamic hypopituitarism secondary to a suprasellar metastasis from a primary small cell lung cancer and review ten cases of hypothalamic hypopituitarism due to metastasis, including our patient.

## Case presentation

A 67-year-old Japanese man with a past medical history of hypertension, diabetes mellitus, and angina presented with a history of generalized weakness, lethargy, cold intolerance, weight loss, and loss of appetite. The patient was a smoker who had been smoking a half-pack per day for 47 years. His family history was unremarkable.

One and one-half months prior to admission, the patient had symptoms of lethargy and anorexia, with a 7-kg weight loss in only 1 month. On the admission day, he could not move because of overall weakness and lethargy and was transferred to our hospital. He was conscious and oriented, and his blood pressure was low (104/70 mmHg) compared with his previous hypertension. His blood glucose on arrival was low (64 mg/dl). His body temperature was 35.8 °C, heart rate (HR) was 60 beats/min, and respiratory rate was 20 breaths/min. No conjunctival pallor or thyromegaly was appreciated. Cardiac and pulmonary examination results were normal, other than a positive tilt test. His neurological examination revealed that his higher cognitive functions were normal, as were the cranial pairs, with no visual defect.

Laboratory studies revealed that the patient’s complete blood count and coagulation were normal. Biochemistry tests revealed a sodium level of 134 mEq/L (reference range, 135–147) and hypoglycemia, but the other electrolytes were within normal limits. Notably, the level of thyroid-stimulating hormone (TSH) was 0.505 μIU/ml (reference range, 0.38–4.31), that of free thyroxine (FT4) was 0.61 ng/dl (reference range, 0.82–1.63), and that of free triiodothyronine was 1.67 ng/dl (reference range, 2.17–3.34) (*see* Table 1). An electrocardiogram showed a sinus bradycardiac rhythm (HR, 53 beats/min) with no conduction or repolarization abnormalities. The echocardiogram revealed no abnormalities. We suspected hypopituitarism based on the patient’s hypoglycemia, hypotension, nonelevated TSH, and low FT4, and also based on the results of head computed tomography (CT) and magnetic resonance imaging (MRI).

**Table 1:** Summary of the laboratory data on admission

		Reference range
Complete blood count		
White blood cells (× 10^2^/μl)	66	33–97
Hemoglobin (g/dl)	14.2	13.1–17.6
Hematocrit (%)	42.4	38.1–50.8
Mean cell volume (fl)	88.7	84.6–100.6
Platelet counts (× 10^4^/μl)	17.6	12.4–30.5
Coagulation		
PT-INR	1.22	0.89–1.12
APTT (s)	39.0	23.6–31.3
Biochemistry		
Total bilirubin (mg/dl)	1.2	0.1–1.2
Aspartate aminotransferase (IU/L)	44	12–35
Alanine aminotransferase (IU/L)	24	6–40
Lactate dehydrogenase (IU/L)	298	119–229
γ-Glutamyl transpeptidase (IU/L)	31	0–48
Blood urea nitrogen (mg/dl)	10.6	7.4–19.5
Creatinine (mg/dl)	0.91	0.5–1.2
Total protein (g/dl)	6.7	6.4–8.3
Albumin (g/dl)	3.7	3.8–5.2
Na (mEq/L)	134	135–147
K (mEq/L)	4.1	3.4–4.8
Cl (mEq/L)	98	98–110
Glucose (mg/dl)	64	70–110
HbA1c (%)	6.6	4.6–6.2
C-reactive protein (mg/dl)	1.61	0–0.5
Thyroid-stimulating hormone (μIU/ml)	0.51	0.38–4.31
Free triiodothyronine (pg/ml)	1.67	2.17–3.34
Free thyroxine (ng/dl)	0.61	0.82–1.63

*APTT* Activated partial thromboplastin time, *PT-INR* Prothrombin time international normalized ratio

**Fig. 1:**
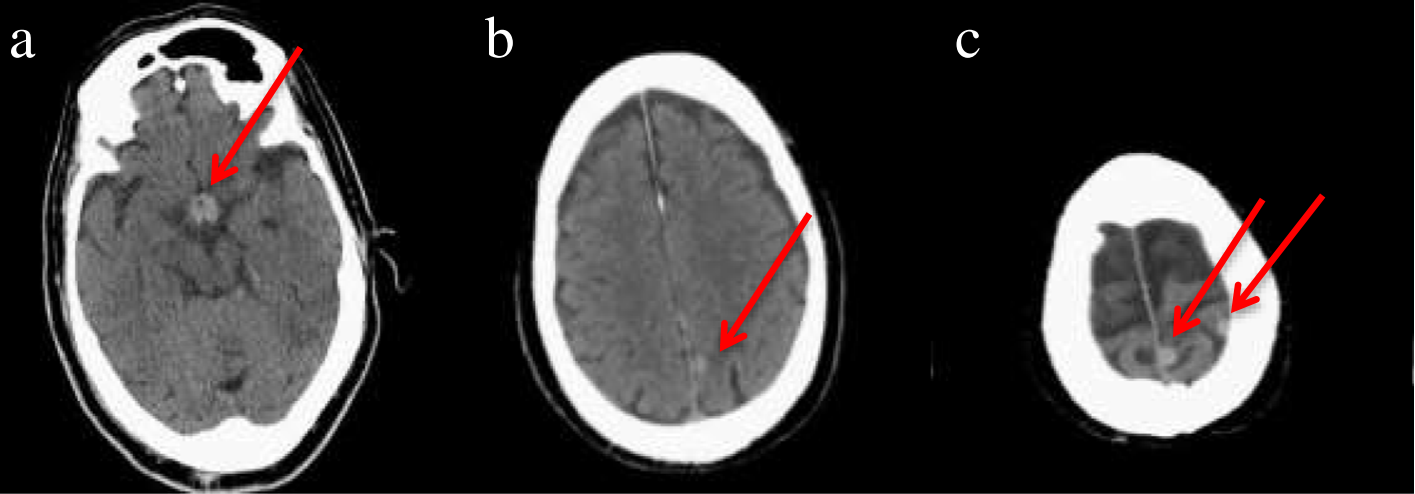
Head computed tomographic scan demonstrating a high-density area on the pituitary (**a**) and multiple tumor-like lesions pointed to by the arrows (**a**–**c**)

**Fig. 2:**
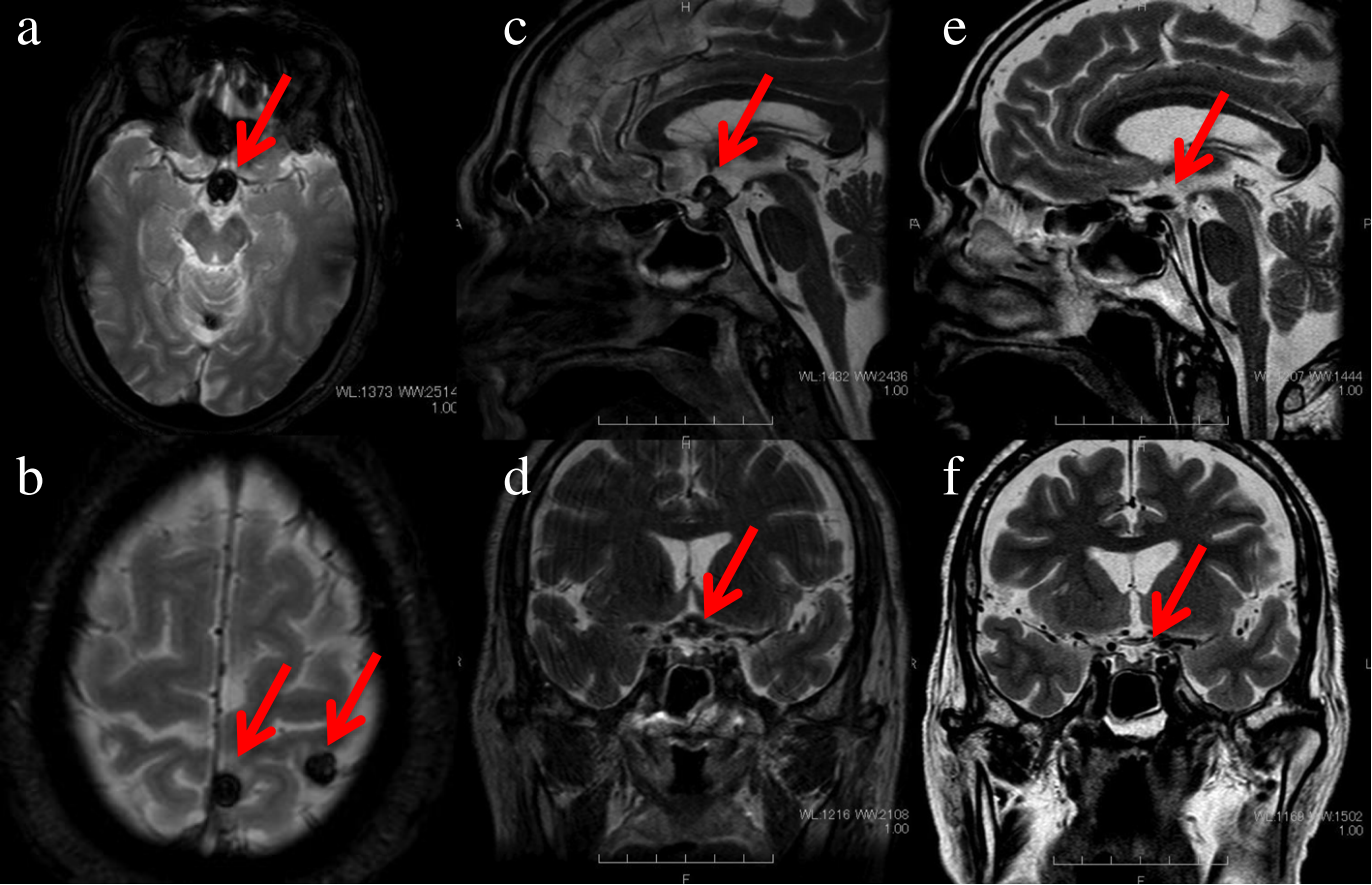
Brain magnetic resonance imaging (MRI) in T2 star-weighted sequences revealing multiple masses (**a**, **b**) and a 10-mm mass on the recessus infundibulum (**c**, **d**). On day 62, brain MRI in T2 star-weighted sequences demonstrated the disappearance of the suprasellar tumor (**e**, **f**) The arrows are pointing to multiple masses

**Fig. 3:**
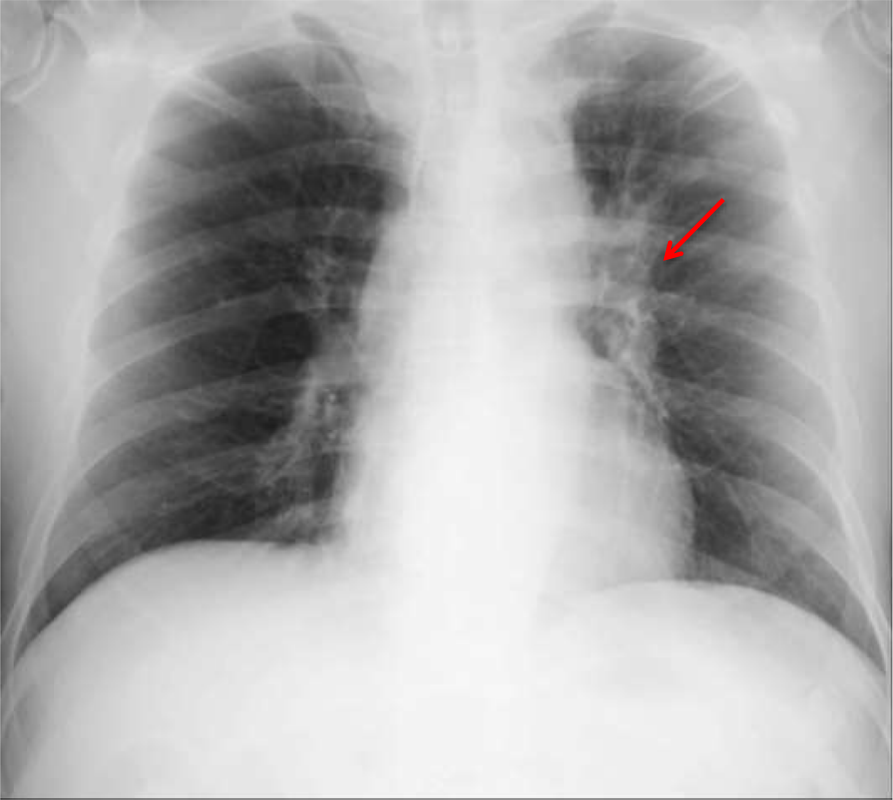
Chest x-ray revealing a left hilar mass (*red arrow*)

**Fig. 4:**
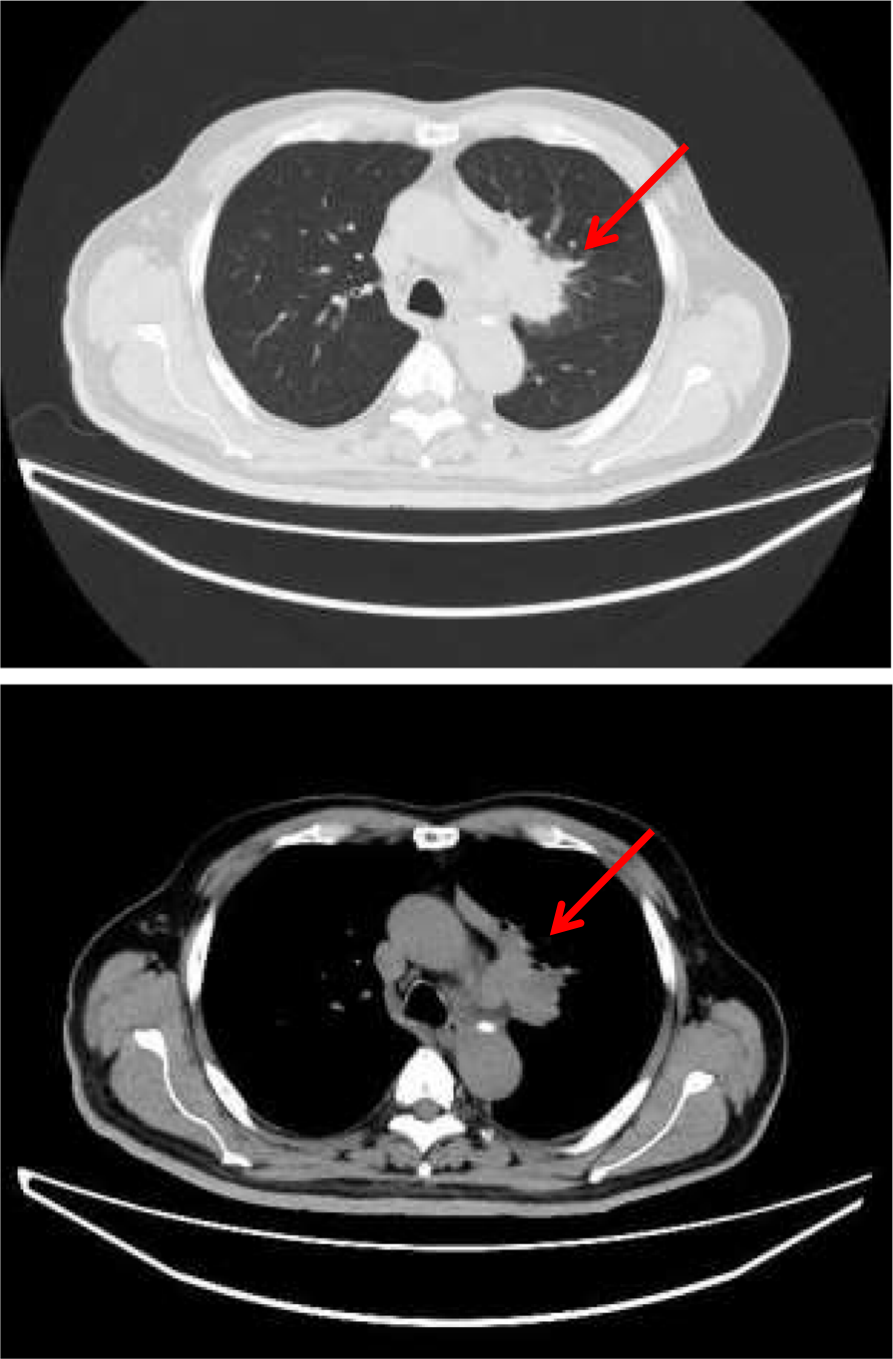
Chest computed tomographic scan revealing a mass in the left hilum (*red arrow*)

CT of the head demonstrated a high-density area in the pituitary-hypothalamic axis, which suggested a hemorrhagic lesion (Fig. 1). Subsequently, MRI of the brain was performed, revealing multiple masses (Fig. 2a, b) and a 10-mm mass on the recessus infundibulum in T2 star-weighted sequences, which were also consistent with hemorrhagic masses (Fig. 2c, d). In addition, a chest x-ray revealed a left hilar mass (Fig. 3). We also noted a suspicious shadow in the left lung hilum shown in the chest radiograph. Subsequently, CT of the thorax revealed a mass in the left hilum (Fig. 4), which was confirmed on biopsy by bronchoscopy later. There were no emphysematous changes on the chest CT scan, but some mediastinal lymphadenopathies were detected.

**Fig. 5:**
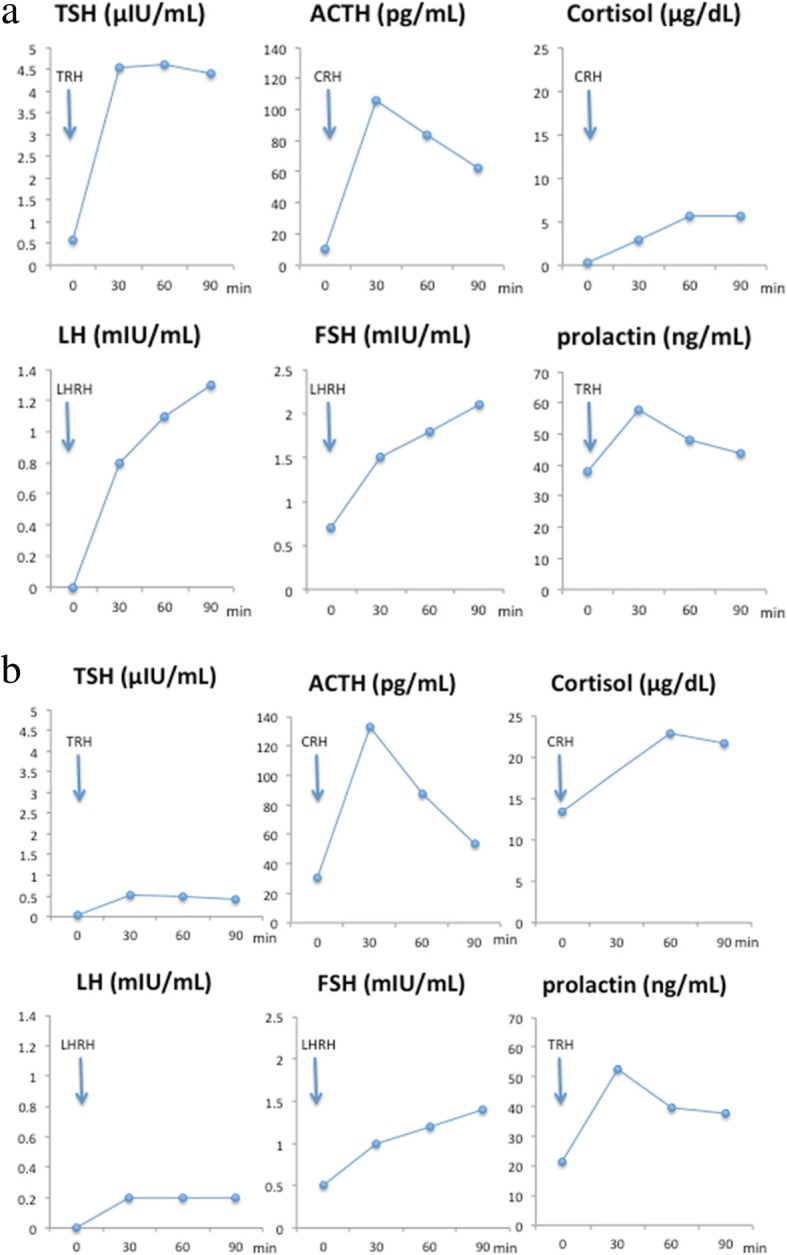
Hormone load test on day 4 (**a**) and on day 59 (**b**)

After the patient’s admission, we first performed a hormone loading test on day 4. The results of the hormone profile at baseline were as follows: TSH 0.586 μIU/ml, follicle-stimulating hormone (FSH) 0.7 mIU/ml (reference range, 2–8.3), luteinizing hormone (LH) < 0.1 mIU/ml (reference range, 1.2–7.1), prolactin 37.9 ng/ml (reference range, 3.6–12.8), testosterone < 4.3 ng/dl (reference range, 142.4–923.1), adrenocorticotropic hormone (ACTH) 5.6 pg/ml (reference range, 7.2–63.3), and cortisol 0.2 μg/dl (reference range, 4.5–21.1). The results showed a TSH response to thyrotropin-releasing hormone: serum TSH 4.526 μIU/ml at 30 min and 4.591 μIU/ml at 60 min. We demonstrated an adequate cortisol and ACTH response to corticotropin-releasing hormone: serum cortisol was 5.6 μg/ dl at 60 min and had a peak of 5.7 μg/ dl at 90 min, whereas serum ACTH showed a peak of 106.3 pg/ml at 30 min, 84.0 pg/ml at 60 min, and 62.4 pg/ml at 90 min. It also showed a delayed LH and FSH response to LH-releasing hormone: serum LH showed a value of 0.8 mIU/ml at 30 min, 1.1 mIU/ml at 60 min, and peak of 1.3 mIU/ml at 90 min, whereas serum FSH was 1.5 mIU/ml at 30 min, 1.8 mIU/ml at 60 min, and peak of 2.1 mIU/ml at 90 min (Fig. 5a). Those results suggested hypothalamic pan-hypopituitarism.

**Fig. 6:**
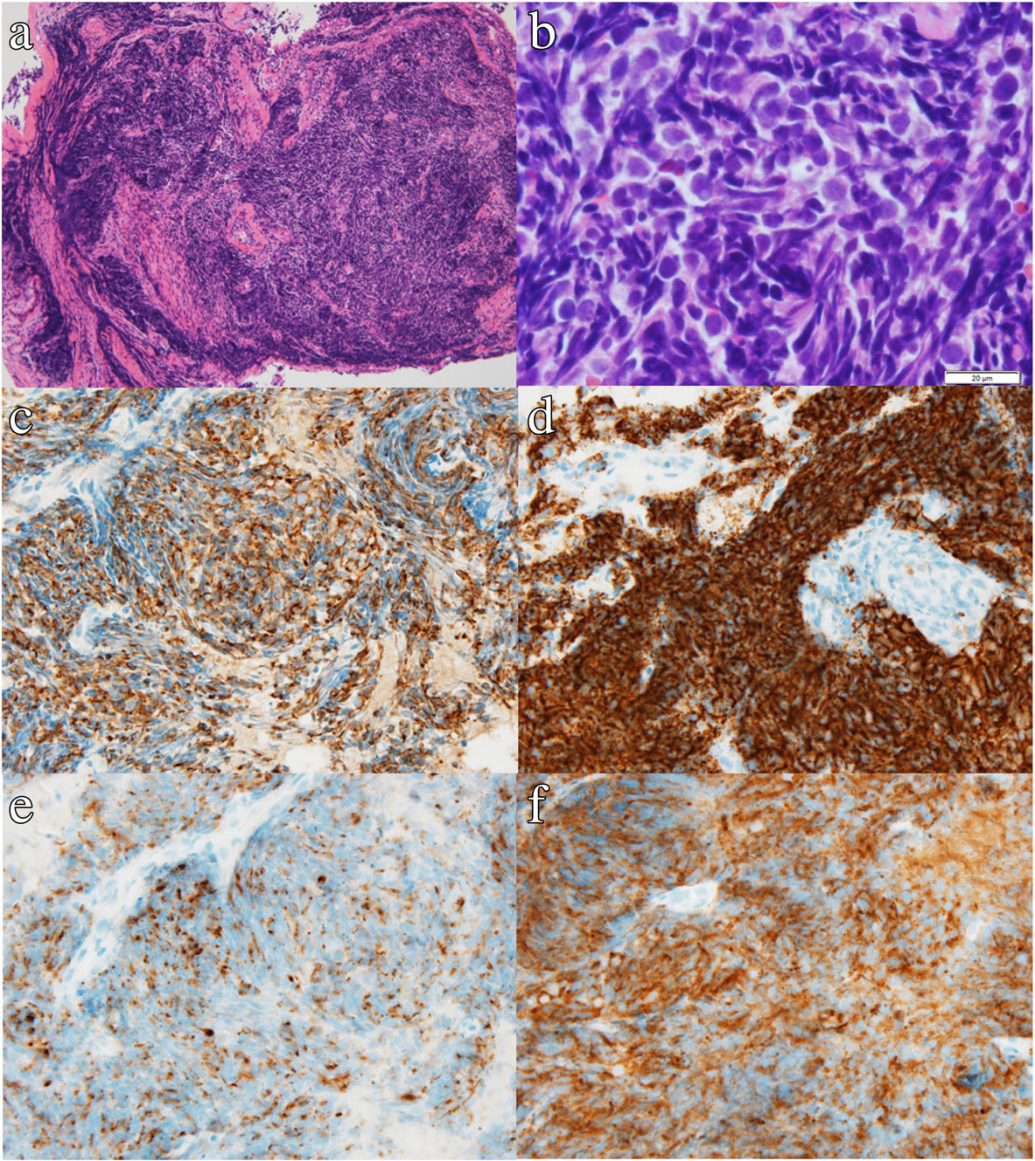
Pathological results obtained from the bronchoscopic biopsy indicating a small cell lung cancer. Hematoxylin and eosin stain shows sheets, rosettes, or peripheral palisading of small-sized round cells with minimal cytoplasm. Densely packed small tumor cells were reactive for cytokeratin (AE1/AE3), CD56, chromogranin, and synaptophysin antibodies immunohistochemically. **a** Hematoxylin and eosin stain, × 100 magnification. **b** Hematoxylin and eosin stain, × 1000 magnification. **c** AE1/AE3 immunostain, × 400 magnification. **d** CD56 immunostain, × 400 magnification. **e** Chromogranin immunostain, × 400 magnification. **f** Synaptophysin immunostain, × 400 magnification

Tumor markers demonstrated elevated pro-gastrin-releasing peptide with a value of 678 pg/ml (reference range, 0–80.0) and neuron-specific enolase 18.6 ng/ml (reference range, 0–12), which are typical markers for small cell lung cancer. We performed lung biopsy by bronchoscopy. Endobronchial ultrasound of bronchoscopy was used, and transbronchial lung biopsies were performed. The pathological results eventually revealed a small cell lung cancer (Fig. 6).

We made a diagnosis of pan-hypopituitarism secondary to suprasellar metastases from a small cell lung cancer and first initiated hormone replacement therapy with hydrocortisone and levothyroxine. Right after the replacement therapy, the symptoms of lethargy, loss of appetite, and hypotension were improved. There was no evidence of diabetes insipidus during those therapies. The patient’s clinical stage was stage IV (cT3N2M1b), and his performance status was 0 to 1; thus, he was treated with adjuvant cranial radiotherapy on day 24 and chemotherapy with cisplatin and etoposide on day 45. We reevaluated the hormone loading test on day 59 after admission, which revealed an improvement in ACTH and cortisol secretions (Fig. 5b). We also performed a brain MRI in T2 star-weighted sequences again on day 62, which demonstrated disappearance of the suprasellar tumor (Fig. 2e, f). The patient was subsequently treated with four cycles of chemotherapy, but he died 10 months later due to the progression of the lung cancer. An autopsy was not performed, because the patient’s family denied permission.

## Discussion

We report a case of a 67-year-old man with a history of smoking who presented with generalized weakness, lethargy, and weight loss and was later diagnosed with hypothalamic hypopituitarism secondary to metastases from the primary lung cancer. He underwent radiation therapy followed by chemotherapy and hormone replacement therapy, but he died 10 months later. What is unique to our patient’s case compared with previously reported cases is that hypothalamic hypopituitarism was confirmed by a hormone loading test, and we performed it again to assess the therapeutic effects. Due to limited literature in regard to cases of hypothalamic hypopituitarism, it was challenging to develop an evidence-based treatment. However, our case was successful because the period between the first visit and the initiation of therapy was within 1 month, although the metastasis itself suggested a poor prognosis, and we could not control the progression of the systemic symptoms.

Metastasis to the pituitary gland is a very rare condition observed in approximately 5% of patients with cancer, although approximately two-thirds of them are latent [1]. Breast cancer is the most common primary malignancy metastasizing to the pituitary. The frequency of breast cancer (39.7%) in the primary tumor metastases to the pituitary is followed by lung cancer (23.7%) [1]. Recognition of hypopituitarism can be challenging, especially in the elderly, whose symptoms such as lethargy and visual decline may be mistaken for the natural aging process. In patients with an established metastatic disease, the symptoms may be wrongly attributed to malignancy or to the side effects of therapy [3]. The systemic complications of malignancy, including nonspecific symptoms (weakness, vomiting, and weight loss) and central nervous system involvement, may mask the anterior pituitary deficiency [4]. McCormick *et al.* reviewed the location of metastasis to the pituitary in 201 cases and reported the posterior lobe either alone or in combination with the anterior lobe in 84.6%, whereas only the anterior lobe was seen in 15.4% [1, 5]. The tendency for metastasis to occur to the posterior lobe is due to the lack of direct arterial blood supply to the anterior lobe, whereas the posterior lobe is supplied by the hypophyseal arteries. For these reasons, the most common symptom of hypopituitarism due to metastasis seems to be diabetes insipidus [6]. The presence of visual loss and anterior pituitary insufficiency, though common symptoms of pituitary adenoma, are less commonly seen with pituitary metastasis [7].

There are two main types of hypopituitarism according to the pituitary or hypothalamic lesions. Even though pituitary hypopituitarism is relatively rare, only ten cases of hypothalamic hypopituitarism secondary to metastases of solid tumors (including our patient) have been reported in English-language journals [8–11] (*see* Table 2). The rate is higher in men than in women; six of the ten reported cases occurred in males. The average age at presentation is 54 (range, 37–76) years. The primary disease in five of the ten cases was lung cancer, whereas four of those were breast cancer. The remaining case was stomach cancer. This is partially consistent with the epidemiology of metastases to the pituitary gland, although the frequency of breast cancer and that of lung cancer were opposite that in the previous review we mentioned above [1].

**Table 2:** Clinical features of hypothalamic hypopituitarism secondary to metastasis reported in the literature

Patient	Reference	Age (years)	Sex	Predisposing factor	Primary disease	Symptoms	Diabetes insipidus	Metastases	Therapy	Outcome
1	8	50	Female	Breast cancer	Breast cancer	Lethargy, body weight gain, memory loss	No	Bone	Hormone replacement therapy	Died some weeks later
2	9	58	Male	Heavy cigarette smoker, alcohol abuse	Small cell lung cancer	Lethargy, body weight loss, decreased libido, diarrhea, cold intolerance	Yes	Cerebral, liver, adrenal grand	Hormone replacement therapy, radiation, chemotherapy	Died 9 month later
3	10	73	Male	Heavy cigarette smoker (1pack for 50 years)	Small cell lung cancer	Lethargy, loss of appetite, neck swelling, cough	Yes	Cerebral	Hormone replacement therapy, radiation, chemotherapy	Died 10 month later
4	11	47	Male	Not described	Stomach cancer	Diabetes insipidus	Yes	Abdominal node, cerebral	Not described	Not described
5	11	52	Female	Not described	Breast cancer	Diabetes insipidus	Yes	Liver	Not described	Not described
6	11	54	Female	Not described	Breast cancer	Diabetes insipidus	Yes	Lung	Not described	Not described
7	11	76	Male	Not described	Lung cancer	Hypopituitarism	Yes	Cerebral	Not described	Not described
8	11	37	Female	Not described	Breast cancer	Diabetes insipidus	Yes	Bone	Not described	Not described
9	11	60	Male	Not described	Lung cancer	Neurologic deficit, hypoadrenalism	No	Cerebral	Not described	Not described
10	Our case	67	Male	Diabetes mellitus, hypertension, angina, heavy smoker (a half a pack for 47 years)	Small cell lung cancer	Lethargy, loss of appetite, body weight loss, generalized weakness, cold intolerance	No	Cerebral	Hormone replacement therapy, radiation, chemotherapy	Died 10 month later

The chief complaints were nonspecific symptoms; the most common were lethargy in four cases, body weight change in three cases, and loss of appetite in two cases. Those symptoms are sometimes associated with the natural aging process, and they may make it harder to reach a true diagnosis. In addition, those symptoms are often confused with the presentations of cancer itself. In addition, diabetes insipidus was present in seven cases. The most significant underlying condition was heavy cigarette smoking, which was reported in three cases, all of which were accompanied by lung cancer.

When hypothalamic hypopituitarism is present, radiotherapy or chemotherapy should be initiated with the least possible delay. The high mortality rate is associated with reported cases of hypothalamic hypopituitarism, and the survival time after diagnosis was at most 10 months, with one case having a survival time of weeks, whereas the others had an unknown outcome. Our case was successful because the period between the first visit and the initiation of therapy was within 1 month, although the metastasis itself suggested a poor prognosis, and we could not control the progression of the systemic symptoms.

## Conclusions

In summary, hypothalamic hypopituitarism is a very rare condition, and the diagnosis is difficult because of the nonspecific symptoms. When the patient is suspected of having hypopituitarism, a hormone load test can help to diagnose the type of hypopituitarism. It is also important to evaluate the brain and the whole body to confirm whether metastasis and primary cancer exist. Because the mortality rate is very high, aggressive intervention for both diagnosis and therapy is required in cases of hypothalamic hypopituitarism secondary to the metastasis.

## Abbreviations

ACTH: Adrenocorticotropic hormone
CT: Computed tomography
FSH: Follicle-stimulating hormone
FT4: Free thyroxine
HR: Heart rate
LH: Luteinizing hormone
MRI: Magnetic resonance imaging
TSH: Thyroid-stimulating hormone
